# Early nerve fibre regeneration in individuals with type 1 diabetes after simultaneous pancreas and kidney transplantation

**DOI:** 10.1007/s00125-019-4897-y

**Published:** 2019-06-07

**Authors:** Shazli Azmi, Maria Jeziorska, Maryam Ferdousi, Ioannis N. Petropoulos, Georgios Ponirakis, Andrew Marshall, Uazman Alam, Omar Asghar, Andrew Atkinson, Wendy Jones, Andrew J. M. Boulton, Michael Brines, Titus Augustine, Rayaz A. Malik

**Affiliations:** 1 Institute of Cardiovascular Sciences, University of Manchester and Central Manchester NHS Foundation Trust, Core Technology Facility, Grafton Street, Manchester, M13 9NT UK; 2 Department of Medicine, Weill Cornell Medicine-Qatar, Education City, Qatar Foundation, Doha, Qatar; 3 Department of Clinical Neurophysiology, Central Manchester NHS Foundation Trust, Manchester, UK; 4 Department of Eye and Vision Science, Institute of Ageing and Chronic Disease, University of Liverpool, Liverpool, UK; 5 Araim Pharmaceuticals, Tarrytown, NY USA; 6 Department of Transplant and Endocrine Surgery, Central Manchester University Hospital NHS Foundation Trust, Manchester, UK

**Keywords:** Corneal confocal microscopy, Diabetic neuropathy, Pancreas and kidney transplantation, Skin biopsy

## Abstract

**Aims/hypothesis:**

The study aimed to assess the impact on neuropathy of simultaneous pancreas and kidney transplantation (SPK) in individuals with type 1 diabetes.

**Methods:**

This longitudinal observational study examined neuropathic symptoms, deficits, quantitative sensory testing, neurophysiology, corneal confocal microscopy and skin biopsy results in 32 healthy (non-diabetic) control participants, 29 individuals with type 1 diabetes and severe diabetic peripheral neuropathy [DPN] and 36 individuals with type 1 diabetes after SPK.

**Results:**

Following SPK, HbA_1c_, eGFR, triacylglycerols and HDL improved significantly (all *p* < 0.05). Compared with the DPN group, which remained unchanged over the 36 month study period, corneal confocal microscopy assessments improved over 36 months following SPK, with increasing corneal nerve fibre density of 5/mm^2^ (95% CI 1.8, 8.2; *p* = 0.003) and corneal nerve fibre length of 3.2 mm/mm^2^ (95% CI 0.9, 5.5; *p* = 0.006). The Neuropathy Symptom Profile and peroneal nerve conduction velocity also improved significantly by 36 months compared with DPN (2.5; 95% CI 0.7, 4.3; *p* = 0.008 and 4.7 m/s; 95% CI 2.2, 7.4; *p* = 0.0004, respectively), but with a temporal delay compared with the corneal confocal microscopy assessments. Intraepidermal nerve fibre density did not change following SPK; however, mean dendritic length improved significantly at 12 (*p* = 0.020) and 36 (*p* = 0.019) months. In contrast, there were no changes in the Neuropathy Disability Score, quantitative sensory testing or cardiac autonomic function assessments. Except for a small decrease in corneal nerve fibre density in the healthy control group, there were no changes in any other neuropathy measure in the healthy control or DPN groups over 36 months.

**Conclusions/interpretation:**

SPK is associated with early and maintained small nerve fibre regeneration in the cornea and skin, followed by an improvement in neuropathic symptoms and peroneal nerve conduction velocity.

**Electronic supplementary material:**

The online version of this article (10.1007/s00125-019-4897-y) contains peer-reviewed but unedited supplementary material, which is available to authorised users.

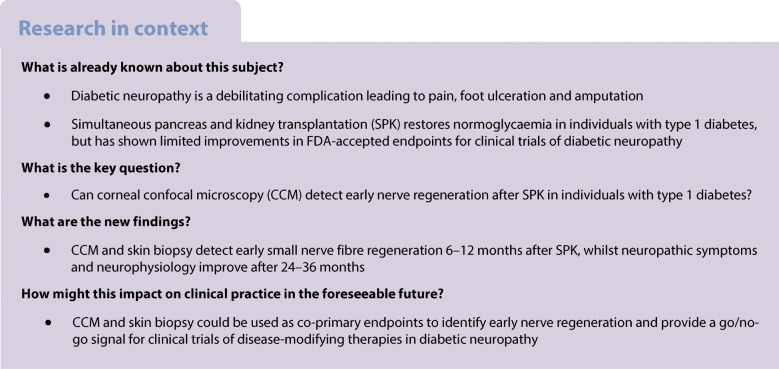



## Introduction

Diabetic neuropathy is a major long-term complication of diabetes, which is associated with pain, foot ulceration and increased mortality. There is currently no Food and Drug Administration (FDA)-approved disease-modifying therapy for diabetic neuropathy. Hyperglycaemia is associated with the development and progression of diabetic neuropathy [1, 2]. Therefore, data from interventions improving or normalising blood glucose may be instructive in the choice of endpoints and the design of clinical trials of new disease-modifying therapies for diabetic neuropathy.

The Epidemiology of Diabetes Interventions and Complications (EDIC) study and the Diabetes Control and Complications Trial (DCCT) showed that intensive glycaemic control reduced the progression of diabetic neuropathy [1]. However, normalisation of blood glucose with simultaneous pancreas and kidney transplantation (SPK) showed no improvement in neurological deficits or autonomic function and an initial improvement in sensory nerve conduction at 12 months was not maintained at 24 or 42 months [3]. A larger study with a 10 year follow-up also demonstrated no impact of SPK on neurological deficits or autonomic function, but there was an improvement in neurophysiology [4]. Recent studies have also shown no significant improvement in neurological disability, nerve conduction, autonomic function or intraepidermal nerve fibre density (IENFD) 2.5 and 8 years after successful SPK [4–6]. It has been argued that the repair of nerves is not possible in individuals undergoing SPK as they have a severe neuropathy. However, we previously used corneal confocal microscopy (CCM) and showed a significant improvement in corneal nerve fibre density (CNFD) and length 6 months after SPK [7]. Subsequently, we showed a significant improvement in CNFD, branch density and length, without a significant improvement in neurological disability, quantitative sensory testing, autonomic function, neurophysiology or IENFD, 12 months after SPK [8]. It was difficult to reconcile the significance of the early improvement in corneal nerve morphology without an improvement in the currently accepted FDA endpoints for clinical trials of diabetic neuropathy.

We have had the unique opportunity to assess neuropathy over 3 years, utilising CCM and skin biopsy, alongside FDA-accepted endpoints, in individuals undergoing SPK [9].

## Methods

### Participant selection

We assessed 36 individuals with type 1 diabetes and end stage renal failure undergoing SPK, 29 individuals with type 1 diabetes and severe diabetic peripheral neuropathy (DPN) and 32 healthy (non-diabetic) control participants from Central Manchester and Manchester Children’s University Hospital. Exclusion criteria were a history of neuropathy due to a non-diabetic cause and any history of corneal trauma or surgery, or systemic or ocular disease that may affect the cornea. The Central Manchester Research and Ethics Committee approved this study and written informed consent was obtained from all participants. This research adhered to the tenets of the declaration of Helsinki.

### Clinical and metabolic assessment

Evaluations were undertaken at baseline, prior to hospital discharge after SPK, and at 6, 12, 24 and 36 months. The control groups were evaluated at the same time points, except 6 months. Study participants underwent assessment of BMI, BP, HbA_1c_, lipid profile (total cholesterol, LDL-cholesterol, HDL-cholesterol and triacylglycerols) and eGFR.

### Neuropathy assessment

Symptoms of DPN were assessed using the Neuropathy Symptom Profile (NSP). Neurological deficits were evaluated using the modified Neuropathy Disability Score (NDS). Vibration perception threshold (VPT) was tested using a Neurothesiometer (Horwell, Scientific Laboratory Supplies, Wilford, Nottingham, UK). Cold (CDT) and warm (WDT) detection thresholds were assessed on the foot using the TSA-II NeuroSensory Analyser (Medoc, Ramat-Yishai, Israel). Sural sensory nerve amplitude, conduction velocity and latency, and peroneal motor nerve amplitude, conduction velocity and latency were determined by a consultant neurophysiologist using a Dantec ‘Keypoint’ system (Dantec Dynamics, Bristol, UK). Heart rate variability was assessed with an ANX 3.0 autonomic nervous system-monitoring device (ANSAR Medical Technologies, Philadelphia, PA, USA).

### Skin biopsy

Three millimetre punch skin biopsies were obtained from the dorsum of the foot, approximately 2 cm proximal to the second metatarsal head, under local anaesthesia (1% lidocaine) at baseline (*n* = 12), 12 months (*n* = 12) and 36 months (*n* = 5) after SPK. Fifty micrometre sections were immunostained using anti-human protein gene product 9.5 (PGP9.5) antibody (Abcam, Cambridge, UK) and nerve fibres were demonstrated using SG chromogen (Vector Laboratories, Peterborough, UK). Growth associated protein-43 (GAP-43), a marker found in newly regenerated nerve fibres [10], was immunolocalised using anti-human GAP-43 antibody (Novus Biologicals, Abingdon, UK). The biopsies were assigned a coded number and all nerve morphology assessments were performed blinded to the case diagnosis. IENFD was quantified according to established international guidelines and expressed as number/mm [11]. IENFD reflects the number of nerves crossing the epidermal basal membrane into the epidermis and does not account for morphology of the nerve fibres inside the epidermis. Therefore, additional morphological measures of the intraepidermal nerve fibre were quantified. Mean dendritic length (MDL) assessment was performed on PGP9.5-stained sections according to previously described methods [12–14]. The images were captured on a Zeiss AxioImager 2 microscope and Z-stack constructs of six sections were obtained (Axiovision and ZEN lite programmes, Carl Zeiss Microimaging, Jena, Germany). The MDL is the mean value of all of the main nerve fibres from the point of their penetration through the basement membrane to their terminal portion and is expressed as μm [14]. Total nerve fibre length (TNFL) assessment was performed on Z-stack constructs of six adjacent GAP-43-stained sections. TNFL is the sum of all nerve fibre profiles in the epidermis and is expressed as μm/mm^2^. A recent study has shown that TNFL can identify nerve fibre regeneration in a clinical trial of cibinetide in individuals with small fibre neuropathy due to sarcoidosis [15].

### Corneal Confocal Microscopy

Participants underwent examination with CCM (Heidelberg Retinal Tomograph III Rostock Cornea Module, Heidelberg Engineering, Heidelberg, Germany) according to our established protocol [16]. A minimum of six non-overlapping images per individual (three per eye) from the centre of the cornea were selected and quantified in a masked fashion. Data derived from these images were averaged for each eye and the mean of both eyes used in subsequent data analysis. Four corneal nerve variables were quantified: CNFD, the total number of major nerves/mm^2^ of corneal tissue; corneal nerve branch density (CNBD), the number of branches emanating from the major nerve trunks/mm^2^; corneal nerve fibre length (CNFL), the total length of all nerve fibres and branches (mm/mm^2^) within the area of corneal tissue; and corneal nerve fibre area (CNFA), the total area of the nerve fibre net (μm^2^/mm^2^). CNFA is a two-dimensional measure which accounts for the length and variable thickness of the corneal nerve fibre bundles. Analysis of corneal nerve morphology was performed using automated software (ACCMetrics for CNFD, CNBD and CNFL, and FIJI for CNFA), as previously described [15].

### Statistical analysis

Statistical analyses were carried out using SPSS for Mac (Version 19.0, IBM Corporation, New York, NY, USA) and JMP (Version 11, SAS, Cary, NC, USA). All data contained within Tables 1 and 2 are expressed as mean ± SD. Plots show least squares mean ± SEM. The data were first assessed for normality using the Shapiro–Wilk normality test. Longitudinal change from baseline value for each study variable was estimated using a mixed model repeated measures analysis, which included participant group, time point and group by time point as fixed effects and participant as a random effect. Least mean squares differences within groups were assessed, as well as the difference between the SPK and DPN groups. Using data published in previous studies, a sample size of 21 participants was calculated to have 90% power to detect within-patient change of 4 nerves per mm^2^ in CNFD. This assumes a 5% significance level, and that the SD of the within-subject differences is 5.5.

**Table 1:** Demographic and biochemical data of participant groups

Variable	Group	Baseline	6 months	12 months	24 months	36 months
Age (years)	SPK	48.6 ± 9.2	–	–	–	–
	DPN	61.9 ± 12.3	–	–	–	–
	HC	47.7 ± 1.6				
Sex (F/M)	SPK	11/25	–	–	–	–
	DPN	13/16	–	–	–	–
	HC	13/17	–	–	–	–
Smoking (cigarettes/day)	SPK	1.9 ± 3.4	–	–	–	–
	DPN	2.1 ± 5.1	–	–	–	–
	HC	0.4 ± 1.9	–	–	–	–
Alcohol consumption (units/week)	SPK	6.6 ± 9.9	–	–	–	–
	DPN	4.7 ± 7.8	–	–	–	–
	HC	2.8 ± 5.4	–	–	–	–
Duration of diabetes (years)	SPK	32.3 ± 10.5	–	–	–	–
	DPN	46.0 ± 13.9	–	–	–	–
	HC	–	–	–	–	–
BP (mmHg)						
Systolic	SPK	131.8 ± 22.3	130.7 ± 4.2	130.7 ± 4.1	127.6 ± 5.7	123.2 ± 5.0
	DPN	141 ± 23.9	–	141.4 ± 27.6	137.1 ± 24.9	132.5 ± 18.0
	HC	129.5 ± 19	–	128.4 ± 15.5	127.1 ± 13.2	129.7 ± 10.2
Diastolic	SPK	73.9 ± 10.5	70.9 ± 2.6	72.7 ± 2.3	68.5 ± 2.0	64.7 ± 2.7
	DPN	72.8 ± 9.8	–	72.2 ± 19.1	68.8 ± 9.8	63.8 ± 8.9
	HC	72.3 ± 10	–	70.3 ± 8.5	72.2 ± 7.2	71.9 ± 10.2
BMI (kg/m^2^)	SPK	23.6 ± 5.3	–	25.3 ± 0.9	25.3 ± 0.8	26.2 ± 1.3
	DPN	27.1 ± 3.6	–	26.8 ± 0.8	27.1 ± 0.7	26.5 ± 0.7
	HC	27.9 ± 4.4	–	27.4 ± 5.1	27.1 ± 6.1	27.3 ± 4.9
HbA_1c_ (%)	SPK	8.4 ± 1.6	5.7 ± 0.9*	5.6 ± 1.1*	5.6 ± 0.9*	5.4 ± 0.7*
	DPN	8.3 ± 1.3	–	8.3 ± 1.4	8.9 ± 1.9	8.0 ± 1.3
	HC	5.7 ± 0.6	–	5.5 ± 0.4	5.5 ± 0.5	5.5 ± 3.0
HbA_1c_ (mmol/mol)	SPK	67.9 ± 16.9	38.4 ± 10.5*	37.6 ± 11.6*	38.3 ± 10.2*	39.0 ± 7.6*
	DPN	66.7 ± 13.4	–	67.5 ± 14.8	68.3 ± 17.8	65.1 ± 14.8
	HC	38.5 ± 3.3	–	37.1 ± 3.1	36.3 ± 3.2	37.1 ± 2.9
eGFR (ml min^−1^ [1.73 m]^−2^)	SPK	14.6 ± 2.4	57.8 ± 4.9*	58.3 ± 4.8*	60.3 ± 4.2*	63.9 ± 5.1*
	DPN	77.1 ± 20.6	–	73.1 ± 18.3	72.4 ± 17.0	75.2 ± 15.7
	HC	83.5 ± 1.8	–	80.2 ± 9.6	83.1 ± 7.1	80.7 ± 8.6
Cholesterol (mmol/l)	SPK	4.2 ± 0.9	4.1 ± 0.2	3.9 ± 1.0	4.1 ± 1.1	4.0 ± 0.9
	DPN	4.3 ± 0.8	–	4.2 ± 0.8	4.0 ± 0.8	4.0 ± 0.7
	HC	5.0 ± 1.5	–	4.9 ± 0.1	5.0 ± 0.6	5.1 ± 0.7
HDL-cholesterol (mmol/l)	SPK	1.5 ± 0.5	1.5 ± 0.1	1.4 ± 0.5	1.5 ± 0.5	1.8 ± 0.8*
	DPN	1.7 ± 0.6	–	1.8 ± 0.4	1.7 ± 0.4	1.7 ± 0.3
	HC	1.5 ± 0.6	–	1.6 ± 0.3	1.5 ± 0.4	1.5 ± 0.4
LDL-cholesterol (mmol/l)	SPK	2.1 ± 0.7	2.1 ± 0.8	2.1 ± 0.9	2.1 ± 0.9	1.8 ± 0.6
	DPN	2.1 ± 0.6	–	2.0 ± 0.6	1.8 ± 0.5	1.8 ± 0.6
	HC	3.0 ± 0.6	–	2.8 ± 0.5	2.8 ± 0.5	2.9 ± 0.6
Triacylglycerols (mmol/l)	SPK	1.2 ± 0.4	1.2 ± 0.1	1.1 ± 0.5	1.1 ± 0.3	0.8 ± 0.3*
	DPN	1.1 ± 0.6	–	0.9 ± 0.4	1.0 ± 0.5	0.9 ± 0.3
	HC	1.4 ± 1.4	–	1.4 ± 1.1	1.5 ± 0.8	1.4 ± 0.7

Data are mean ± SD

*p < 0.05, **p < 0.01 compared with baseline

F, female; HC, healthy control; M, male

**Table 2:** Neuropathy assessments over time in the study groups

Variable	Group	Baseline	6 months	12 months	24 months	36 months
NDS	SPK	5.2 ± 3.7	5.3 ± 3.8	5.6 ± 3.6	3.1 ± 3.6	4.7 ± 3.7
	DPN	4.1 ± 3.5	–	5.4 ± 3.2	5.4 ± 3.2	4.0 ± 3.3
	HC	0.3 ± 0.8	–	0.2 ± 0.5	0.2 ± 0.9	0.2 ± 0.5
NSP	SPK	5.3 ± 5.9	4.6 ± 6.6	5.1 ± 6.8	3.5 ± 7.5	2.5 ± 4.2*
	DPN	6.0 ± 7.0	–	6.2 ± 6.1	7.2 ± 7.8	6.0 ± 7.4
	HC	0.4 ± 1.1	–	0.4 ± 1.0	0.1 ± 0.4	0.1 ± 0.1
VPT (V)	SPK	22.1 ± 13.5	19.5 ± 10.7	21.1 ± 14.3	19.1 ± 12.5	22.8 ± 17.1
	DPN	21.6 ± 13.6	–	23.3 ± 13.9	23.3 ± 13.9	23.7 ± 14.6
	HC	4.9 ± 3.4	–	6.3 ± 8.7	5.2 ± 3.1	4.4 ± 1.4
Sural amplitude (μV)	SPK	3.6 ± 2.5	3.8 ± 2.6	4.4 ± 2.0	5.6 ± 1.9	6.9 ± 2.4
	DPN	6.2 ± 6.4	–	5.7 ± 5.7	5.3 ± 4.9	5.9 ± 5.9
	HC	19.8 ± 9.8	–	21.3 ± 8.3	21.1 ± 8.6	20.2 ± 7.5
Sural velocity (m/s)	SPK	34.4 ± 7.9	36.3 ± 8.7	37.2 ± 8.6	37.0 ± 8.9	37.8 ± 8.2
	DPN	38.7 ± 7.8	–	37.7 ± 7.8	36.4 ± 7.2	37.3 ± 8.5
	HC	51.0 ± 4.6	–	50.5 ± 4.4	50.3 ± 5.5	50.3 ± 5.0
Peroneal amplitude (mV)	SPK	1.4 ± 1.3	1.5 ± 1.4	1.6 ± 1.3	1.4 ± 1.2	1.8 ± 0.7
	DPN	2.4 ± 1.9	–	1.8 ± 176	2.4 ± 1.9	2.8 ± 1.9
	HC	5.8 ± 2.0	–	6.0 ± 1.7	5.3 ± 1.7	5.8 ± 1.8
Peroneal velocity (m/s)	SPK	36.1 ± 5.1	37.9 ± 4.9	37.8 ± 6.3	38.0 ± 7.5*	40.9 ± 3.5**
	DPN	37.4 ± 8.7	–	36.9 ± 8.5	37.0 ± 8.9	37.0 ± 8.8
	HC	49.2 ± 3.4	–	49.0 ± 3.3	48.3 ± 5.0	48.8 ± 4.8
CDT (°C)	SPK	16.0 ± 11.1	18.0 ± 11.0	16.8 ± 12.2	17.1 ± 12.1	17.6 ± 12
	DPN	23.5 ± 8.2	–	22.3 ± 8.2	22.3 ± 8.2	23.1 ± 7.6
	HC	27.8 ± 5.5	–	28.4 ± 2.3	27.1 ± 4.3	28.3 ± 1.6
WDT (°C)	SPK	43.8 ± 4.9	44.3 ± 4.3	43.4 ± 4.9	42.8 ± 5.1	40.9 ± 4
	DPN	40.8 ± 4.7	–	41.5 ± 4.4	41.5 ± 4.4	42.2 ± 5.1
	HC	35.2 ± 6.8	–	38.3 ± 3.4	38.0 ± 3.5	37.1 ± 2.5
DB-HRV (beats/min)	SPK	14.6 ± 14.9	10.1 ± 6.0	10.1 ± 7	10.6 ± 9.5	11.3 ± 7.4
	DPN	10.0 ± 8.2	–	6.7 ± 8.9	15.3 ± 10.6	16.9 ± 8.4
	HC	33.2 ± 10.1	–	31.7 ± 13.6	29.4 ± 13.0	30.5 ± 14.0
CNFD (number/mm^2^)	SPK	9.2 ± 5.8	11.7 ± 6.2*	12.2 ± 8.4**	12.5 ± 6.6**	14.4 ± 5.0**
	DPN	11.4 ± 5.6	–	12.6 ± 8.1	11.6 ± 6.9	9.0 ± 5.5
	HC	30.8 ± 7.3	–	31.8 ± 3.9	28.9 ± 6.0	28.2 ± 5.7*
CNBD (number/mm^2^)	SPK	9.7 ± 8.4	10.3 ± 8.3	13.2 ± 13.2	12.7 ± 8.9	15.0 ± 6.6*
	DPN	10.6 ± 7.7	–	11.4 ± 8.6	10.6 ± 8.8	7.9 ± 4.8
	HC	40.0 ± 17.3	–	43.7 ± 16.3	39.7 ± 13.3	36.3 ± 15.2*
CNFL (mm/mm^2^)	SPK	7.1 ± 3.0	7.3 ± 2.7	8.2 ± 4.0	8.9 ± 3.5*	10.3 ± 2.0**
	DPN	7.8 ± 2.8	–	9.2 ± 4.0	8.2 ± 3.3	7.6 ± 2.5
	HC	17.8 ± 3.5	–	18.6 ± 2.5	17.1 ± 3.0	16.8 ± 3.0
CNFA (μm^2^/mm^2^)	SPK	10,867 ± 4410	11,554 ± 4537	12,746 ± 6629	13,743 ± 5419*	15,394 ± 4893**
IENFD (number/mm)	SPK	2.2 ± 2.5	–	2.9 ± 2.8	–	2.9 ± 1.9
	DPN	5.2 ± 4.4	–	–	3.4 ± 2.9	–
	HC	9.6 ± 2.8	–	–	9.5 ± 1.8	–
MDL (μm)	SPK	10.9 ± 2.6	–	15.6 ± 4.8*	–	18.0 ± 3.0**
TNFL (μm/mm^2^)	SPK	2797 ± 3651	–	3954 ± 4927	–	2881 ± 2545

Data are mean ± SD

*p < 0.05, **p < 0.01 compared with baseline

HC, healthy control

## Results

### SPK improves metabolic variables

At baseline, the control, DPN and SPK cohorts did not differ significantly in age, sex or alcohol consumption (Table 1). The SPK group had a significantly higher HbA_1c_ (*p* < 0.001) and lower eGFR (*p* < 0.001), total cholesterol (*p* < 0.001) and LDL-cholesterol (*p* < 0.001) compared with control participants. Following SPK, HbA_1c_ and eGFR improved significantly (*p* < 0.05). Additionally, there was a significant reduction in triacylglycerols (*p* < 0.05) and increase in HDL (*p* < 0.05) (Table 1).

### Participants with type 1 diabetes have a severe neuropathy prior to SPK

At baseline, the SPK group exhibited marked small and large fibre abnormalities compared with the control cohort. Neuropathic symptoms (NSP), deficits (NDS), quantitative sensory thresholds (VPT, WDT, CDT), cardiac autonomic function (deep breathing heart rate variability [DB-HRV]) and electrophysiology (sural and peroneal nerve amplitude and velocity) were significantly abnormal compared with healthy control participants (Table 2) (all *p* < 0.001). CNFD, CNBD, CNFL and CNFA on CCM and IENFD on skin biopsy were significantly lower at baseline in the SPK group compared with control participants (all *p* < 0.001) (Table 2).

### No change in neuropathy assessments in control participants and participants with type 1 diabetes and severe neuropathy over 36 months

**Fig. 1:**
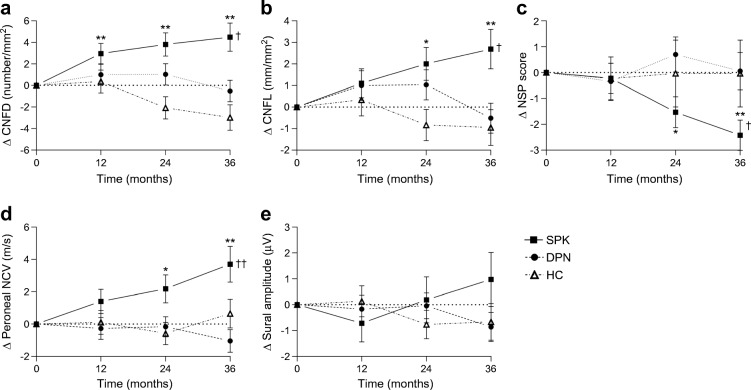
Longitudinal least mean squares change from baseline values confirms early and sustained improvement in (**a**) CNFD and (**b**) CNFL, with a later improvement in (**c**) NSP score, (**d**) peroneal NCV and (**e**) sural nerve amplitude. *p < 0.05, **p < 0.01 vs baseline; ^†^*p* < 0.01, ^††^*p* < 0.001 SPK vs DPN over the time course; all statistical comparisons by mixed model repeated measures analysis. HC, healthy control; NCV, nerve conduction velocity

**Fig. 2:**
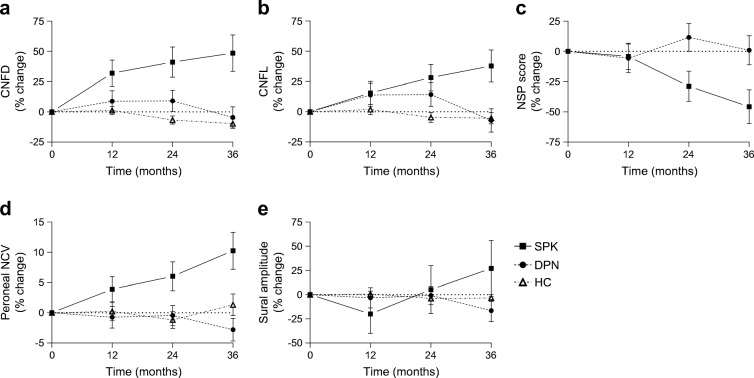
Percentage change from baseline values in (**a**) CNFD, (**b**) CNFL, (**c**) NSP score, (**d**) peroneal NCV and (**e**) sural nerve amplitude. HC, healthy control; NCV, nerve conduction velocity

There was no significant change in NSP, NDS, VPT, CDT, WDT, DB-HRV, sural and peroneal nerve amplitude and velocity, CNBD, CNFL, or IENFD in the healthy control and DPN groups over 36 months (Table 2). There was a decrease in CNFD of 3.0 ± 1.3 fibres/mm^2^ (least squares mean; *p* = 0.024) (Fig. 1a), which was an ~10% decrease from baseline, at 36 months in control participants (Fig. 2a).

### SPK is associated with early and maintained small nerve fibre regeneration

**Fig. 3:**
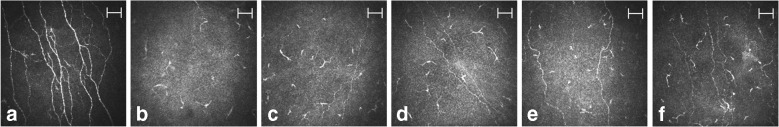
Corneal confocal images of the sub-basal nerve plexus in (**a**) a control participant and (**b**–**f**) an SPK patient at baseline (**b**) and at 6 (**c**), 12 (**d**), 24 (**e**) and 36 (**f**) months, showing small fibre regeneration. Scale bars, 50 μm

In the SPK group, corneal nerve fibre regeneration was evident at 6 months and continued over 36 months (Figs. 1a, b and 3). There was a significant improvement in CNFD (Table 2; *p* = 0.035) 6 months after SPK, but no corresponding change in any other measure of neuropathy (Table 2). At 12 months, there was a significant improvement from baseline in CNFD (*p* = 0.007), but no significant change in CNFL, CNFA, NSP, NDS, VPT, CDT, WDT, DB-HRV or neurophysiology (Table 2). Corneal nerve morphology continued to improve over follow-up, such that at 24 months CNFD (*p* < 0.01), CNFL (*p* < 0.05) and CNFA (*p* < 0.05) were significantly increased compared to baseline (Table 2) and at 36 months the least squares mean CNFD of the SPK group had increased by 5 fibres/mm^2^ (95% CI 1.8, 8.2; *p* = 0.003) and CNFL by 3.2 mm/mm^2^ (95% CI 0.9, 5.5; *p* = 0.006) over the DPN group. The relative percentage change from baseline over the 36 month time period for CNFD and CNFL for the SPK group was ~49% and ~38%, respectively, whereas the DPN group decreased by ~5% and ~7%, respectively (Fig. 2a, b).

**Fig. 4:**
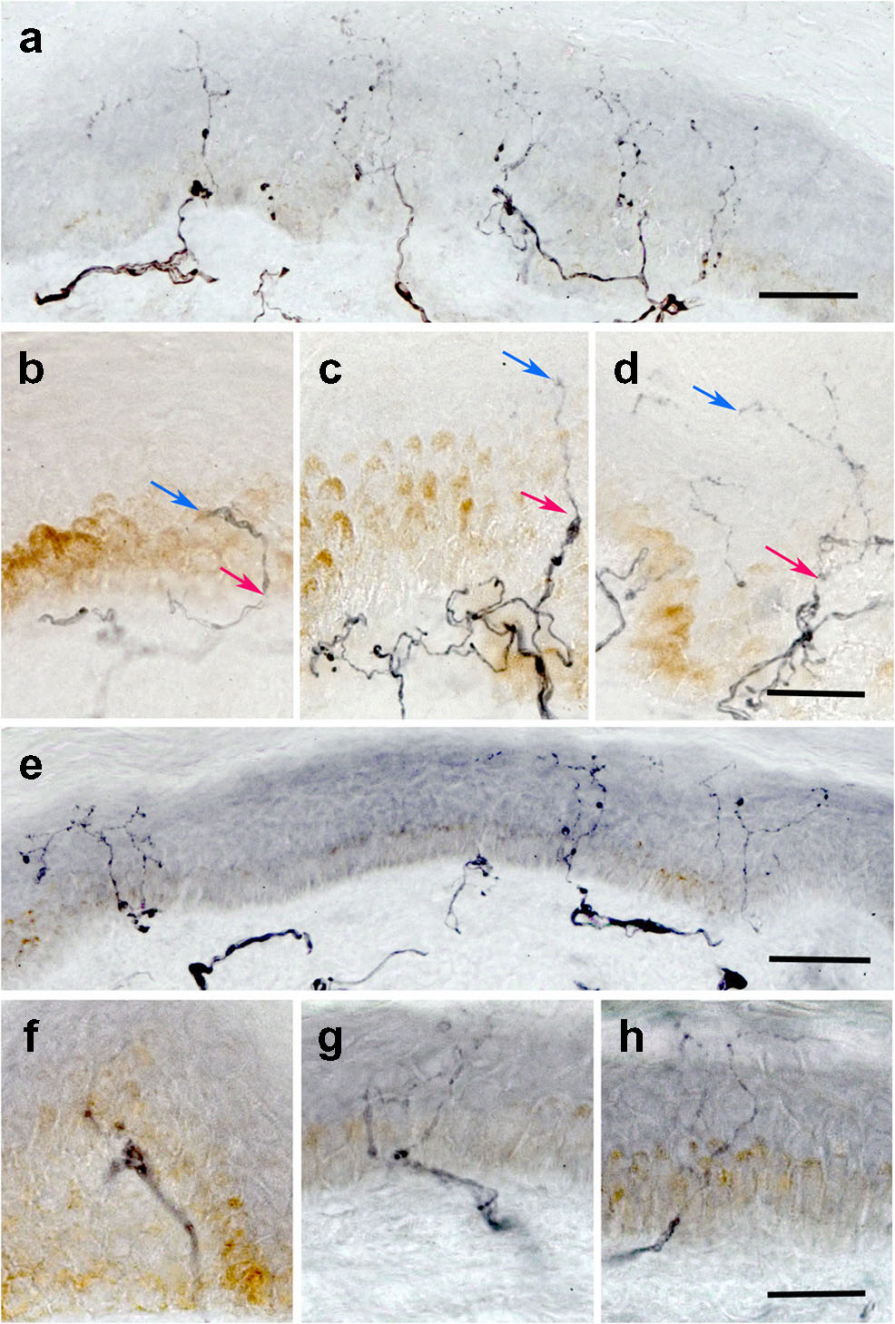
Representative examples of 50 μm skin biopsy sections immunostained for (**a**–**d**) PGP9.5 and (**e**–**h**) GAP-43 from a healthy control participant (**a**, **e**) and an SPK patient (**b**–**d**, **f**–**h**) . (**a**) The healthy control sample shows numerous long branching intraepidermal nerve fibres **(**IENFs) reaching the upper epidermis and a well-developed subepidermal nerve plexus. IENFs in an SPK patient at baseline (**b**) are sparse and short, showing (**c**) elongation and (**d**) branching after 12 months. Red arrows indicate nerves crossing the basement membrane and blue arrows show the terminal part of the IENF. (**e**) GAP-43 immunostained nerve fibres from the same healthy individual as in (**a**) are long and branching and those from an SPK patient at baseline (**f**) and at 12 (**g**) and 24 (**h**) months after SPK show a similar pattern as those stained for PGP9.5. Scale bars in (**a**) and (**e**), 50 μm; scale bars in (**b**–**d**) and (**f**–**h**), 25 μm

A subgroup of SPK participants underwent skin biopsy at baseline (*n* = 12), 12 months (*n* = 12) and 36 months (*n* = 5) (Fig. 4). MDL increased significantly at 12 (*p* = 0.020) and 36 (*p* = 0.019) months, but there was no significant change in IENFD or TNFL (Table 2).

There was a reduction in the number of participants followed from baseline (*n* = 36) to 6 (*n* = 25), 12 (*n* = 24), 24 (*n* = 24) and 36 (*n* = 15) months. However, there was no significant difference in the baseline clinical and neuropathy measures between the individuals who were followed compared with the individuals who were lost to follow-up (Electronic Supplementary Material Table 1).

### SPK is associated with a delayed improvement in symptoms and neurophysiology

The NSP (2.5; 95% CI 0.7, 4.3; *p* = 0.008; Fig. 1c) improved significantly from baseline in the SPK group at 36 months. Similarly, peroneal nerve conduction velocity improved at 36 months compared with baseline (4.7 m/s; 95% CI 2.2, 7.4; *p* = 0.0004; Fig. 1d), with no change in sural nerve amplitude (Fig. 1e). The relative change from baseline of NSP at 36 months was ~ −46% (improvement) for the SPK group compared with ~1% for the DPN group (Fig. 2c). The percentage change from baseline for the peroneal nerve conduction velocity was ~10% for the SPK group compared with ~ −3% for the DPN group (Fig. 2d), compared with sural nerve amplitude changes from baseline of ~27% for the SPK group and ~17% for the DPN group (Fig. 2e).

## Discussion

This study shows that SPK in individuals with type 1 diabetes results in small fibre regeneration of corneal nerve fibres as early as 6 months and intraepidermal nerve fibres at 12 months, followed by an improvement in neuropathic symptoms and neurophysiology by 36 months. However, there were no improvements in neuropathic deficits, vibration and thermal thresholds, cardiac autonomic function or IENFD. Thus, our study shows an early and sustained improvement, but not normalisation, of some measures of small fibre structure following SPK. An early study demonstrated no impact on neurological deficits and autonomic function and an initial improvement in sensory nerve conduction, which was not maintained at 42 months [3]. A small, prospective study of 18 individuals undergoing SPK showed an improvement in median nerve conduction velocity at 48 months [17], but another study showed no improvement in thermal thresholds [18]. The lack of improvement may reflect the subjective variability in quantitative sensory testing, especially in individuals with advanced neuropathy. A landmark study with a follow-up over 10 years showed a significant improvement in nerve conduction studies, but no impact on neurological deficits or autonomic function [4]. Other studies assessing cardiac autonomic function after SPK have also reported conflicting results [19, 20].

With regard to assessing improvements in structural abnormalities, small nerve fibres possess a greater ability to regenerate compared with large nerve fibres [21]. Experimental studies have shown nerve regeneration in rats 15 months after pancreas transplantation [22] and as early as 3 weeks after islet cell transplantation [23]. In the present study, we have utilised novel measures of small nerve fibre morphology to show corneal and intraepidermal nerve fibre regeneration. This supports our previous studies showing that CCM can detect early corneal nerve fibre regeneration within 12 months of SPK [7, 8], after continuous subcutaneous insulin infusion [24] and after treatment with cibinetide [25]. A recent study has also demonstrated a significant 29% improvement in CNFL after 12 months of treatment with omega-3 polyunsaturated fatty acids, with no change in neurophysiology or quantitative sensory testing [26]. In the present study, the improvement in small fibres was associated with HbA_1c_ normalisation and an improvement in HDL and triacylglycerols, which are known risk factors for the development of diabetic neuropathy [27], although a change in neurotrophic support may also have contributed [28]. There was no significant change in any of the measures of neuropathy in control participants, apart from a small reduction in CNFD, which is comparable with a study in 50 healthy control participants followed over 36 months [29]. We also show no significant worsening of neuropathy in individuals with type 1 diabetes and severe neuropathy over 36 months. This is consistent with three recent prospective studies in cohorts of individuals with mild to moderate diabetic neuropathy showing no or minimal deterioration in a range of neuropathy measures [30–32], attributed to relatively good control of risk factors for diabetic neuropathy.

Although IENFD is considered the gold standard for identifying early small nerve fibre damage and repair, two studies have shown no improvement in distal thigh IENFD 2.5 years [5] and 8 years [6] after SPK, and we have also previously shown no change in IENFD 12 months after SPK [8]. We now show that there is no significant improvement in IENFD up to 3 years after SPK. An increase in IENFD requires regeneration of the more proximal nerve fibres of the subepidermal nerve plexus as opposed to the distal intraepidermal nerves, where regeneration will commence. Indeed, in the present study we show that MDL, a measure of more distal intraepidermal nerve regeneration, improves 12 and 36 months after SPK. This suggests that MDL, as opposed to IENFD, may have greater utility in assessing early nerve fibre repair. Furthermore, studies have shown that epidermal axons are capable of regrowth [33, 34], and GAP-43 has been used as an indicator of nerve regeneration [10, 35, 36]. In the present study, the length of intraepidermal nerve fibres expressing GAP-43, expressed as TNFL, also showed a trend for improvement.

A limitation of this study is the small number of individuals assessed, especially those undergoing skin biopsy. However, it is difficult to enrol large numbers of individuals undergoing SPK to undergo extensive neuropathy phenotyping, especially skin biopsy, over a long duration, due to their considerable morbidity and mortality.

In conclusion, individuals with type 1 diabetes show early small nerve fibre regeneration, followed by an improvement in neuropathic symptoms and neurophysiology. This suggests that we need longer clinical trial durations in excess of 3 years when utilising current FDA-accepted endpoints of symptoms, signs and neurophysiology. Alternatively, we argue for the inclusion of CCM and skin biopsy as co-primary endpoints, to provide an early go/no go signal for clinical trials of disease-modifying therapies in diabetic neuropathy.

## Electronic supplementary material

ESM Table1 (PDF 66 kb)

## Data Availability

The datasets generated during and/or analysed during the current study are available from the corresponding author on reasonable request.

## Abbreviations

CCM: Corneal confocal microscopy
CDT: Cold detection threshold
CNBD: Corneal nerve branch density
CNFA: Corneal nerve fibre area
CNFD: Corneal nerve fibre density
CNFL: Corneal nerve fibre length
DB-HRV: Deep breathing heart rate variability
DPN: Diabetic peripheral neuropathy
FDA: Food and Drug Administration
GAP-43: Growth associated protein-43
IENFD: Intraepidermal nerve fibre density
MDL: Mean dendritic length
NDS: Neuropathy Disability Score
NSP: Neuropathy Symptom Profile
PGP9.5: Protein gene product 9.5
SPK: Simultaneous pancreas and kidney transplantation
TNFL: Total nerve fibre length
VPT: Vibration perception threshold
WDT: Warm detection threshold

## References

[1] Albers JW, Herman WH, Pop-Busui R (2010). Effect of prior intensive insulin treatment during the Diabetes Control and Complications Trial (DCCT) on peripheral neuropathy in type 1 diabetes during the Epidemiology of Diabetes Interventions and Complications (EDIC) study. Diabetes Care.

[2] Stratton IM, Adler AI, Neil HA (2000). Association of glycaemia with macrovascular and microvascular complications of type 2 diabetes (UKPDS 35): prospective observational study. BMJ..

[3] Kennedy WR, Navarro X, Goetz FC, Sutherland DE, Najarian JS (1990). Effects of pancreatic transplantation on diabetic neuropathy. N Engl J Med.

[4] Navarro X, Sutherland DE, Kennedy WR (1997). Long-term effects of pancreatic transplantation on diabetic neuropathy. Ann Neurol.

[5] Boucek P, Havrdova T, Voska L (2008). Epidermal innervation in type 1 diabetic patients: a 2.5-year prospective study after simultaneous pancreas/kidney transplantation. Diabetes Care.

[6] Havrdova T, Boucek P, Saudek F (2016). Severe epidermal nerve fiber loss in diabetic neuropathy is not reversed by long-term normoglycemia after simultaneous pancreas and kidney transplantation. Am J Transplant.

[7] Mehra S, Tavakoli M, Kallinikos PA (2007). Corneal confocal microscopy detects early nerve regeneration after pancreas transplantation in patients with type 1 diabetes. Diabetes Care.

[8] Tavakoli M, Mitu-Pretorian M, Petropoulos IN (2013). Corneal confocal microscopy detects early nerve regeneration in diabetic neuropathy after simultaneous pancreas and kidney transplantation. Diabetes..

[9] Malik RA (2016). Wherefore art thou, o treatment for diabetic neuropathy?. Int Rev Neurobiol.

[10] Denny JB (2006). Molecular mechanisms, biological actions, and neuropharmacology of the growth-associated protein GAP-43. Curr Neuropharmacol.

[11] Lauria G, Hsieh ST, Johansson O (2010). European Federation of Neurological Societies/Peripheral Nerve Society Guideline on the use of skin biopsy in the diagnosis of small fiber neuropathy. Report of a joint task force of the European Federation of Neurological Societies and the Peripheral Nerve Society. Eur J Neurol.

[12] Pittenger GL, Ray M, Burcus NI, McNulty P, Basta B, Vinik AI (2004). Intraepidermal nerve fibers are indicators of small-fiber neuropathy in both diabetic and nondiabetic patients. Diabetes Care.

[13] Pittenger GL, Mehrabyan A, Simmons K (2005). Small fiber neuropathy is associated with the metabolic syndrome. Metab Syndr Relat Disord.

[14] Azmi S, Ferdousi M, Petropoulos IN (2015). Corneal confocal microscopy identifies small-fiber neuropathy in subjects with impaired glucose tolerance who develop type 2 diabetes. Diabetes Care.

[15] Culver DA, Dahan A, Bajorunas D (2017). Cibinetide improves corneal nerve fiber abundance in patients with sarcoidosis-associated small nerve fiber loss and neuropathic pain. Invest Ophthalmol Vis Sci.

[16] Tavakoli M, Malik RA (2011) Corneal confocal microscopy: a novel non-invasive technique to quantify small fibre pathology in peripheral neuropathies. J Vis Exp (47):2194 10.3791/2194 PMC3182640 21248693

[17] Solders G, Tyden G, Persson A, Groth CG (1992). Improvement of nerve conduction in diabetic neuropathy. A follow-up study 4 yr after combined pancreatic and renal transplantation. Diabetes..

[18] Navarro X, Kennedy WR, Fries TJ (1989). Small nerve fiber dysfunction in diabetic neuropathy. Muscle Nerve.

[19] Boucek P, Saudek F, Adamec M (2003). Spectral analysis of heart rate variation following simultaneous pancreas and kidney transplantation. Transplant Proc.

[20] Cashion AK, Hathaway DK, Milstead EJ, Reed L, Gaber AO (1999). Changes in patterns of 24-hr heart rate variability after kidney and kidney-pancreas transplant. Transplantation..

[21] Malik RA, Veves A, Walker D (2001). Sural nerve fibre pathology in diabetic patients with mild neuropathy: relationship to pain, quantitative sensory testing and peripheral nerve electrophysiology. Acta Neuropathol.

[22] Orloff MJ, Greenleaf G, Girard B (1990). Reversal of diabetic somatic neuropathy by whole-pancreas transplantation. Surgery.

[23] Schmidt RE, Nelson JS, Johnson EM (1981). Experimental diabetic autonomic neuropathy. Am J Pathol.

[24] Azmi S, Ferdousi M, Petropoulos IN (2015). Corneal confocal microscopy shows an improvement in small-fiber neuropathy in subjects with type 1 diabetes on continuous subcutaneous insulin infusion compared with multiple daily injection. Diabetes Care.

[25] Brines M, Dunne AN, van Velzen M (2015). ARA 290, a nonerythropoietic peptide engineered from erythropoietin, improves metabolic control and neuropathic symptoms in patients with type 2 diabetes. Mol Med.

[26] Lewis EJH, Perkins BA, Lovblom LE, Bazinet RP, Wolever TMS, Bril V (2017). Effect of omega-3 supplementation on neuropathy in type 1 diabetes: a 12-month pilot trial. Neurology..

[27] Tesfaye S, Chaturvedi N, Eaton SE (2005). Vascular risk factors and diabetic neuropathy. N Engl J Med.

[28] Kim HC, Cho YJ, Ahn CW (2009). Nerve growth factor and expression of its receptors in patients with diabetic neuropathy. Diabet Med.

[29] Dehghani C, Pritchard N, Edwards K (2014). Morphometric stability of the corneal subbasal nerve plexus in healthy individuals: a 3-year longitudinal study using corneal confocal microscopy. Invest Ophthalmol Vis Sci.

[30] Dehghani C, Pritchard N, Edwards K (2014). Natural history of corneal nerve morphology in mild neuropathy associated with type 1 diabetes: development of a potential measure of diabetic peripheral neuropathy. Invest Ophthalmol Vis Sci.

[31] Ziegler D, Low PA, Litchy WJ (2011). Efficacy and safety of antioxidant treatment with alpha-lipoic acid over 4 years in diabetic polyneuropathy: the NATHAN 1 trial. Diabetes Care.

[32] Gibbons CH, Freeman R, Tecilazich F (2013). The evolving natural history of neurophysiologic function in patients with well-controlled diabetes. J Peripher Nerv Syst.

[33] Guo G, Kan M, Martinez JA, Zochodne DW (2011). Local insulin and the rapid regrowth of diabetic epidermal axons. Neurobiol Dis.

[34] Cheng C, Guo GF, Martinez JA, Singh V, Zochodne DW (2010). Dynamic plasticity of axons within a cutaneous milieu. J Neurosci.

[35] Fantini F, Johansson O (1992). Expression of growth-associated protein 43 and nerve growth factor receptor in human skin: a comparative immunohistochemical investigation. J Invest Dermatol.

[36] Holahan MR (2017). A shift from a pivotal to supporting role for the growth-associated protein (GAP-43) in the coordination of axonal structural and functional plasticity. Front Cell Neurosci.

