# Supplementary material for: Early nerve fibre regeneration in individuals with type 1 diabetes after simultaneous pancreas and kidney transplantation
Source: Diabetologia. 2019 Jun 7;62(8):1478–87. doi: 10.1007/s00125-019-4897-y (PMC6647173; doi:10.1007/s00125-019-4897-y)
Supplement: Supplementary file 1 — (PDF 66 kb) [file 125_2019_4897_MOESM1_ESM.pdf]

ESM Table 1. Demographic data and Neuropathy Assessment in those who were followed up for 36 months vs those who were lost to follow up.

	Lost to FU at 36 months (n=21)	FU (n=15)	P
**Age (Years)**	44.2±9.9	44.2±8.5	0.8
**Duration of Diabetes (years)**	27.8±11.2	28.3±9.6	0.9
**Blood Pressure systolic/diastolic (mmHg)**	130.9±22.4 / 71.9±12.5	118.9±13.5 / 70.1±7.3	0.07/0.4
**BMI (kg/m^2)**	24.2±3.5	24.2±3.4	0.9
**HbA1c (mmol/mol)**	63.5±9.2	64.4±8.3	0.2
**Cholesterol (mmol/l)**	4.3±0.9	3.8±0.5	0.2
**HDL-C (mmol/l)**	1.4±0.3	1.2±0.2	0.2
**LDL-C (mmol/l)**	2.1±0.7	1.9±0.7	0.3
**Triglycerides (mmol/l)**	1.2±0.4	1.2±0.4	0.7
**NDS**	5.9±3.3	4.6±3.7	0.2
**NSP**	6.7±6.7	4.7±2.7	0.1
**VPT (volts)**	26.1±12.5	19.9±13.3	0.09
**Sural Amplitude (µV)**	2.8±2.8	2.7±2.6	0.9
**Sural Velocity (m/s)**	34.2±8.4	36.2±7.6	0.6
**Peroneal Amplitude (mV)**	1.4±1.2	1.2±1.5	0.6
**Peroneal Velocity (m/s)**	32.4±8.6	31.2±9.6	0.8
**Cold Threshold (˚C)**	13.4±11.4	21.2±8.0	0.1
**Warm Threshold (˚C)**	44.7±4.1	42.7±6.2	0.4
**DB-HRV (beats/min)**	12.2±7.1	14.4±6.4	0.2
**CNFD (no./mm^2)**	9.9±6.3	8.4±5.4	0.6

**CNBD (no./mm^2)**	10.1±8.8	9.0±8.8	0.8
**CNFL (mm/mm^2)**	7.3±2.9	7.3±3.2	1.0
**IENFD (no./mm)**	1.8±2.7	2.7±1.2	0.06
